# CBCT assessment of root and canal morphology in mandibular anterior teeth in a Chilean subpopulation using an alphanumeric coding system

**DOI:** 10.1186/s12903-026-09313-3

**Published:** 2026-07-18

**Authors:** Pablo Betancourt, Camila Saa, Pablo Navarro, Diana Sanz-Serrano, Montse Mercadé, Emmanuel Silva

**Affiliations:** 1 Endodontic Laboratory, Center for Research in Dental Sciences (CICO), Faculty of Dentistry, Universidad de La Frontera, Temuco, Chile; 2 Department of Integral Adultos, Facultad de Odontología, Universidad de La Frontera, Temuco, Chile; 3 Program of Master in Dental Science, Facultad de Odontología, Universidad de La Frontera, Temuco, Chile; 4 Facultad de Ciencias de la Salud, Universidad Autónoma de Chile, Temuco, Chile; 5 Departament of Dentistry, University of Barcelona, Barcelona, Spain; 6 IDIBELL Biomedical Research Institute of Bellvitge, L’Hospitalet de Llobregat, Spain; 7 Department of Endodontics, School of Dentistry, State University of Rio de Janeiro (UERJ), Rio de Janeiro, RJ Brazil; 8 Post-Graduate Program in Translational Biomedicine (BIOTRANS), Grande Rio University , Rio de Janeiro, RJ Brazil; 9 Department of Endodontics, School of Dentistry, Fluminense Federal University (UFF), Niterói, RJ Brazil

**Keywords:** Cone-beam computed tomography, Chile, Endodontics, Root canal, Tooth root

## Abstract

**Background:**

Detailed knowledge of root and canal morphology is essential for successful endodontic treatment. Cone-beam computed tomography (CBCT) allows accurate three-dimensional evaluation of anatomical features and variations.

**Objective:**

To describe the root and canal morphology of 1,008 permanent mandibular anterior teeth in a Chilean subpopulation using CBCT, and to classify canal configurations according to an alphanumeric coding system.

**Methods:**

A total of 168 CBCT scans from patients aged 15–75 years were retrospectively analyzed. The number of roots and canals, root and corono-radicular lengths, and bilateral symmetry were assessed in 336 mandibular central incisors, 336 mandibular lateral incisors, and 336 mandibular canines. Root canal configurations were classified using an alphanumeric coding system.

**Results:**

All mandibular incisors presented a single root (100%), while 1.5% of mandibular canines exhibited two roots. The most common canal configuration was a single canal (^1^ TN ^1−1^), observed in 69.3% of central incisors, 68.4% of lateral incisors, and 90.8% of canines. Complex configurations, including ^1^ TN ^1–2−1^ and ^1^ TN ^2−1^, were also identified. Bilateral symmetry was recorded in 82.7% of central incisors, 82.1% of lateral incisors, and 86.9% of canines.

**Conclusions:**

In this Chilean subpopulation, mandibular anterior teeth predominantly exhibited a single root and a simple canal configuration, with a smaller proportion showing complex anatomies.

## Introduction

The success of endodontic treatment largely depends on a thorough understanding of both the internal and external tooth morphology [1]. Comprehensive knowledge of the anatomy and possible variations of the root canal system is essential for the accurate execution of all stages of endodontic therapy, including chemomechanical preparation, disinfection, and root canal filling. Such understanding helps prevent procedural complications and contributes to improved clinical outcomes [2, 3].

Mandibular anterior teeth - comprising mandibular central incisors, mandibular lateral incisors, and mandibular canines - are typically single-rooted, but may present morphological variations with potential clinical implications in endodontics [4–6]. While most cases exhibit a single canal, occasional anatomical complexities such as additional roots, bifurcations, or multiple canals have been reported in different populations [4–7].

Several morphological classification systems have been proposed to describe the number of roots and root canals [8]. Among these, Vertucci’s classification, established in 1984, has been one of the most widely applied [9, 10]. Its popularity stems from its simplicity and ease of interpretation, making it a practical tool in both clinical and academic settings. Nevertheless, the system includes only a limited range of morphological configurations and may not fully capture the complexity and variability of the root canal system [11, 12]. The development of three-dimensional imaging techniques, such as micro-computed tomography (micro-CT) and cone-beam computed tomography (CBCT), has enabled the identification of anatomical configurations and morphological variations not previously described [11–13]. In this context, Ahmed et al. (2017) proposed an alphanumeric coding system to classify root canal anatomy, aiming to overcome the limitations inherent to two-dimensional studies [11]. This system specifies the tooth number (TN), the number of roots (R), and the configuration of root canals, detailing their course from the orifice (O), through the canal (C), to the apical foramen (F). While highly comprehensive and capable of describing virtually any root canal configuration, its complexity can pose challenges for routine use and may limit straightforward communication among clinicians.

Tooth morphology—both internal and external—is determined not only by the intrinsic anatomical complexity of each tooth, but also by factors such as ethnicity [14], sex [15], age [10], and geographical distribution [4], which can significantly influence the presence and frequency of morphological variations [10]. Despite advances in the understanding of dental morphology worldwide, to the best of our knowledge, no studies have specifically evaluated the internal and external anatomy of permanent mandibular anterior teeth in the Chilean population. Considering the possible morphological differences associated with ethnic and geographic factors, generating local evidence is essential to more precisely guide endodontic clinical practice in this population. Therefore, the aim of this study was to describe the morphology of 1,008 mandibular anterior teeth in a Chilean subpopulation based on images obtained through CBCT, applying this alphanumeric coding system.

## Materials and methods

This cross-sectional, observational, descriptive study was approved by the Scientific Ethics Committee of the Universidad de La Frontera, Temuco, Chile (Protocol No. 095/23). A total of 168 CBCT scans from patients of both sexes (87 females and 81 males) were evaluated. The images were provided by the Radiology Unit of the Faculty of Dentistry, Universidad de La Frontera, Temuco, Chile, and had been acquired between January 2018 and April 2023 as part of routine diagnostic and treatment planning procedures. All patients signed an informed consent to freely participate in this study. Before image analysis, all CBCT scans were anonymized by assigning a unique identification code to each participant and removing all personal identifiers from the dataset. Only age and sex were retained for research purposes. All analyses were performed using the anonymized dataset, ensuring participant confidentiality throughout the study. In all cases, the ALARA (As Low As Reasonably Achievable) principle was strictly applied, using the smallest field of view and the lowest exposure parameters compatible with adequate diagnostic image quality.

The CBCT scans analyzed in this study were selected according to specific inclusion and exclusion criteria. The inclusion criteria comprised scans from patients aged 15 to 75 years with fully developed roots in the mandibular anterior teeth. Exclusion criteria included scans showing intraradicular posts or previous endodontic treatment; teeth restored with fixed prostheses or metallic restorations that could compromise image quality; calcified root canals; radiographic evidence of previous periapical surgery; or developmental dental anomalies that could alter root morphology. The CBCT images were acquired using a Pax-i3D scanner (Vatech, Hwaseong, Korea) with the following parameters: 120 kV, 9 mA, a 12 × 9 cm field of view, and a voxel size of 0.12 mm. A total of 1,008 teeth were analyzed, comprising 336 mandibular central incisors, 336 mandibular lateral incisors, and 336 mandibular canines.

### Image analysis

**Fig. 1:**
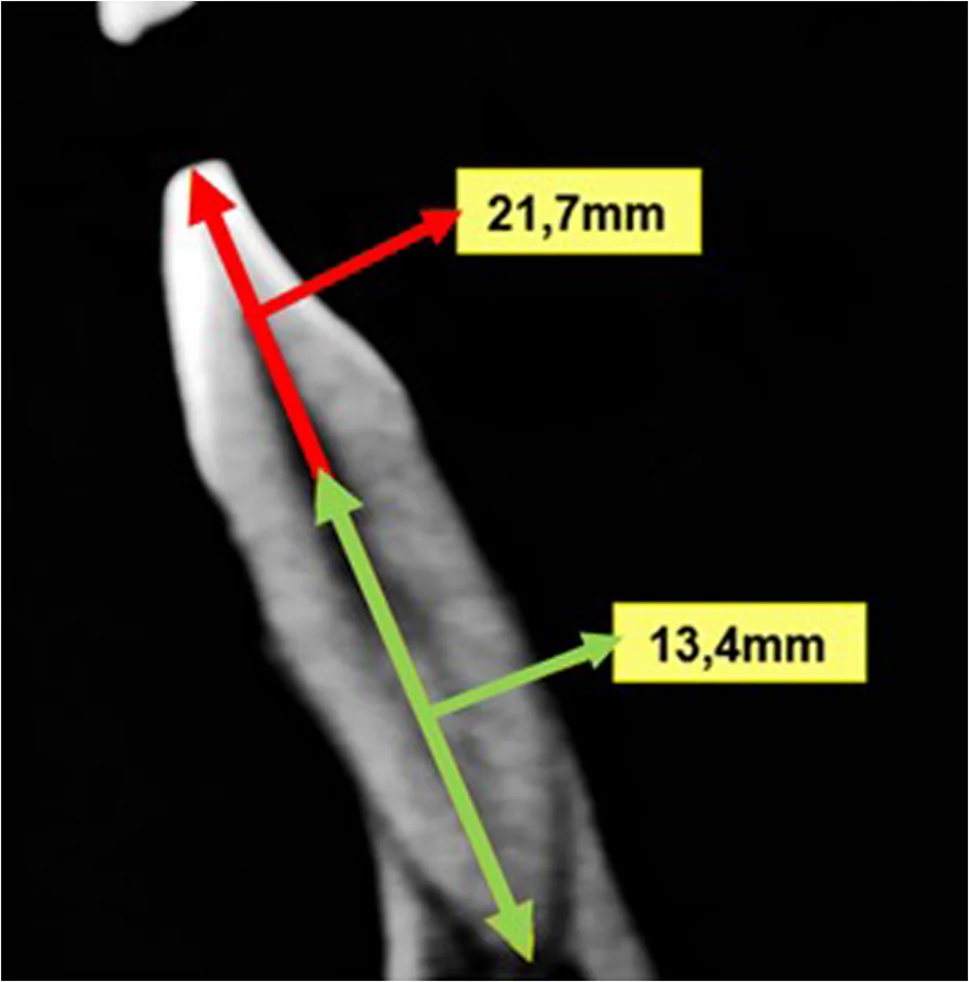
Representative sagittal CBCT image showing the measurement of root length (green arrow) and tooth length (red arrow). Root length was defined as the distance from the cemento-enamel junction (CEJ) to the root apex, whereas tooth length was measured from the root apex to the incisal edge in mandibular central and lateral incisors or to the cusp tip in mandibular canines

**Fig. 2:**
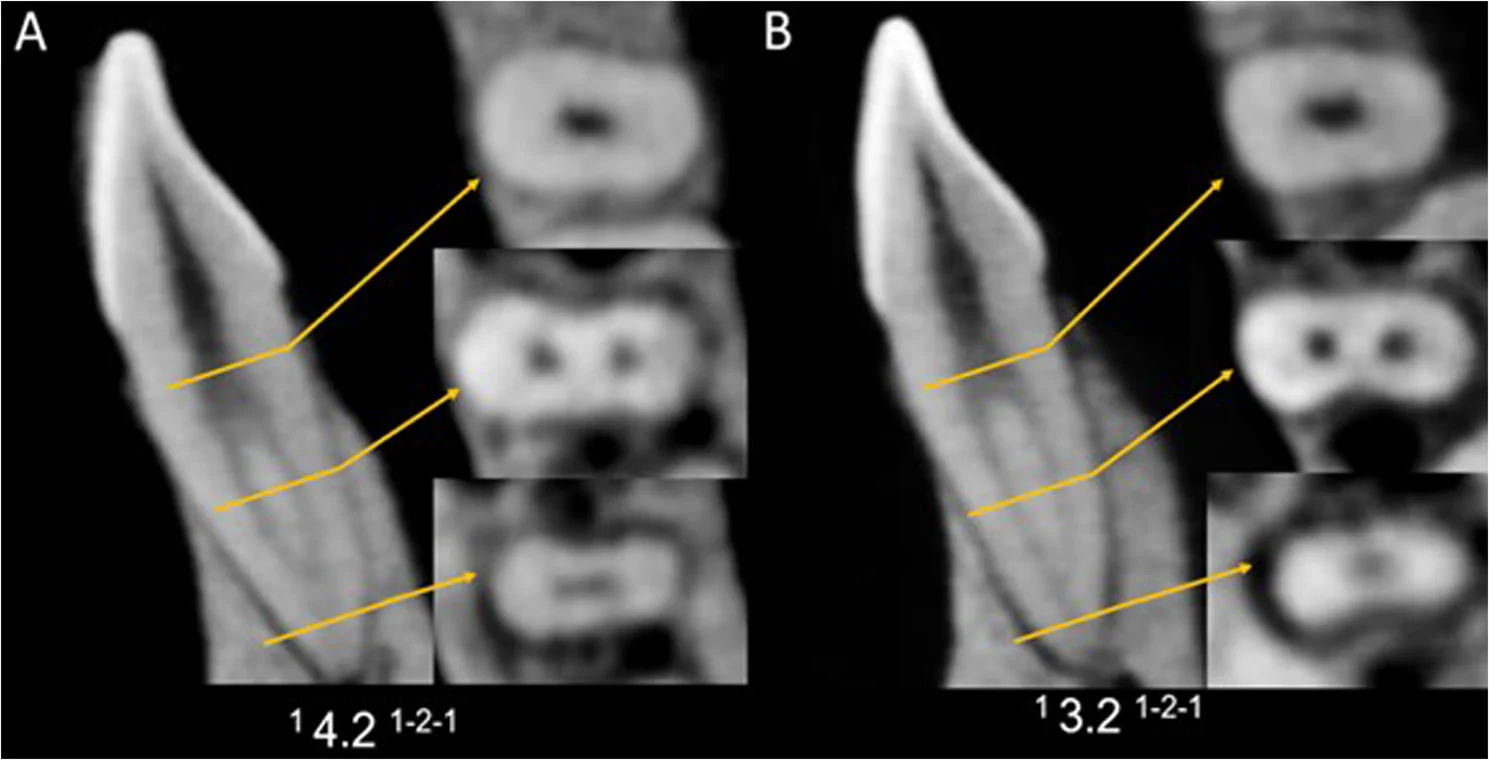
Sagittal and corresponding axial CBCT views of mandibular incisors 4.2 (**A**) and 3.2 (**B**). Both teeth exhibited a single root and a 1–2–1 root canal configuration according to Ahmed’s classification, demonstrating bilateral symmetry (1 4.2 1-2-1 and 1 3.2 1-2-1, respectively). The axial sections at the coronal, middle, and apical thirds illustrate canal bifurcation and subsequent merging along the root canal pathway. Yellow lines indicate the levels at which the axial sections were obtained

The images were processed using Ez3D 2009 software (Vatech, Hwaseong, Korea) and viewed on an LED monitor KDL-42W651A (Sony, Minato, Japan). Multiplanar reconstruction was employed to simultaneously assess axial, sagittal, and coronal slices, enabling localization of the mandibular central incisor (right and left), mandibular lateral incisor (right and left), and mandibular canine (right and left). Root length was measured in the sagittal plane from a perpendicular projection of the cementoenamel junction to the root apex. Tooth length was measured from the root apex to the incisal edge (for mandibular central incisor and mandibular lateral incisor) or to the cusp tip (for mandibular canine) (Fig. 1). The number of roots and root canals, as well as bilateral symmetry between contralateral teeth, were determined through complete corono-apical scanning, evaluating axial slices at 0.5–1.0 mm intervals. Bilateral symmetry was assessed descriptively by comparing the number of roots and root canal configurations between right and left contralateral teeth of the same type. Symmetry was considered present when both teeth exhibited identical anatomical characteristics (Fig. 2). No additional agreement statistics were calculated for this analysis. The root canal system morphology was classified using an alphanumeric coding system previously described (Fig. 2) [11].

Two investigators independently analyzed the images after completing inter-observer calibration. Both examiners were dentists with formal training in Endodontics and previous experience in CBCT image interpretation and root canal morphology assessment. Reliability was assessed using the Kappa concordance coefficient, which indicated a very good level of agreement (κ = 0.886; *p* > 0.05). Calibration consisted of three separate evaluations of 20 CBCT scans of mandibular anterior teeth, with one-week intervals between sessions. In cases of disagreement, a third examiner, an experienced oral and maxillofacial radiologist with expertise in endodontic imaging, reviewed the images and made the final decision by consensus.

### Statistical analysis

Data were entered into a Microsoft Excel spreadsheet and analyzed using IBM SPSS Statistics, version 23.0 (IBM Corp., Armonk, NY, USA). Continuous variables were expressed as means and standard deviations, and categorical variables as frequencies and percentages. Comparisons between groups were performed using the independent Student’s t-test for continuous variables and the chi-square test for categorical variables. The significance level was set at *p* < 0.05.

## Results

A total of 1,008 permanent mandibular anterior teeth were analyzed. All mandibular central incisors and mandibular lateral incisors had a single root (100%), whereas two roots were observed in five cases (1.5%) of mandibular canines — a variation found exclusively in female subjects (Table 1).

**Table 1:** Prevalence of the number of roots in mandibular anterior teeth. Frequency and percentage distribution of root numbers observed in mandibular central incisors, mandibular lateral incisors, and mandibular canines assessed by CBCT

Tooth	Gender	One root	Two roots
Mandibular central incisor	Female	162 (48.2%)	-
	Male	174 (51.8%)	-
	Total	336 (100%)	-
Mandibular lateral incisor	Female	162 (48.2%)	-
	Male	174 (51.8%)	-
	Total	336 (100%)	-
Mandibular canine	Female	157 (46.7%)	5 (1.5%)
	Male	174 (51.8%)	-
	Total	331 (98.5%)	5 (1.5%)

The mean root length on the right side was 12.13 mm for mandibular central incisor, 13.78 mm for mandibular lateral incisor, and 15.43 mm for mandibular canine. On the left side, the values were similar: 12.11 mm for mandibular central incisors, 13.87 mm for mandibular lateral incisors, and 15.31 mm for mandibular canine. Statistically significant differences in root length were observed between sexes (Table 2). Regarding mean tooth length, the measurements on the right side were 20.75 mm for mandibular central incisors, 22.66 mm for mandibular lateral incisors, and 25.49 mm for mandibular canines. On the left side, the corresponding values were 20.72 mm, 22.71 mm, and 25.46 mm, respectively. Statistically significant differences in tooth length between sexes were also observed (Table 2).

**Table 2:** Mean and standard deviation of root and tooth length. Mean values (± standard deviation) of root and tooth lengths (in millimeters) for mandibular central incisors, mandibular lateral incisors, and mandibular canines assessed by CBCT

Tooth	Side	Gender	*n*	Root length (mm)	*p*-value	Tooth length (mm)	*p*-value
Mandibular central incisor	Right	Female	81	11.83 ± 1.13	0.003*	20.44 ± 1.31	0.011*
		Male	87	12.40 ± 1.31		21.04 ± 1.66	
		Total	168	12.13 ± 1.26		20.75 ± 1.53	
	Left	Female	81	11.77 ± 1.29	0.002*	20.39 ± 1.30	0.007*
		Male	87	12.43 ± 1.42		21.04 ± 1.74	
		Total	168	12.11 ± 1.40		20.72 ± 1.57	
Mandibular lateral incisor	Right	Female	81	13.46 ± 1.09	0.002*	22.33 ± 1.23	0.005*
		Male	87	14.09 ± 1.46		22.96 ± 1.66	
		Total	168	13.78 ± 1.33		22.66 ± 1.49	
	Left	Female	81	13.46 ± 1.20	0.001*	22.31 ± 1.28	0.001*
		Male	87	14.25 ± 1.48		23.07 ± 1.73	
		Total	168	13.87 ± 1.41		22.71 ± 1.57	
Mandibular canine	Right	Female	81	14.92 ± 1.80	0.001*	24.54 ± 1.69	0.001*
		Male	87	15.91 ± 1.98		26,38 ± 2,01	
		Total	168	15.43 ± 1.95		25.49 ± 2.07	
	Left	Female	81	14.81 ± 1.84	0.001*	24.51 ± 2.02	0.001*
		Male	87	15.78 ± 1.80		26.35 ± 1.96	
		Total	168	15.31 ± 1.88		25.46 ± 2.19	

In terms of root canal configuration, two canals were observed in 103 mandibular central incisors (30.7%), 106 mandibular lateral incisors (31.5%), and 31 mandibular canines (9.2%). Bilateral symmetry in root canal morphology was found in 139 mandibular central incisors (82.7%), 138 mandibular lateral incisors (82.1%), and 146 mandibular canines (86.9%) (Table 3).

**Table 3:** Cases with bilaterally symmetrical root canal numbers. Frequency and percentage of bilateral symmetry in the number and configuration of root canals in mandibular anterior teeth (mandibular central incisors, mandibular lateral incisors, and mandibular canines). Symmetry was considered when right and left teeth of the same type presented identical numbers and configurations of root canals

Teeth	Bilaterally symmetrical root canal	Total
Mandibular central incisor	139 (82.7%)	168
Mandibular lateral incisor	138 (82.1%)	168
Mandibular canine	146 (86.9%)	168

According to the alphanumeric coding system, the ^1^ TN ^1−1^ configuration was the most prevalent, occurring in 233 mandibular central incisors (69.3%), 230 mandibular lateral incisors (68.4%), and 300 mandibular canines (89.3%). The ^1^ TN ^1–2−1^ configuration was found in 86 mandibular central incisors (25.6%), 88 mandibular lateral incisors (26.2%), and 15 mandibular canines (4.5%).

**Fig. 3:**
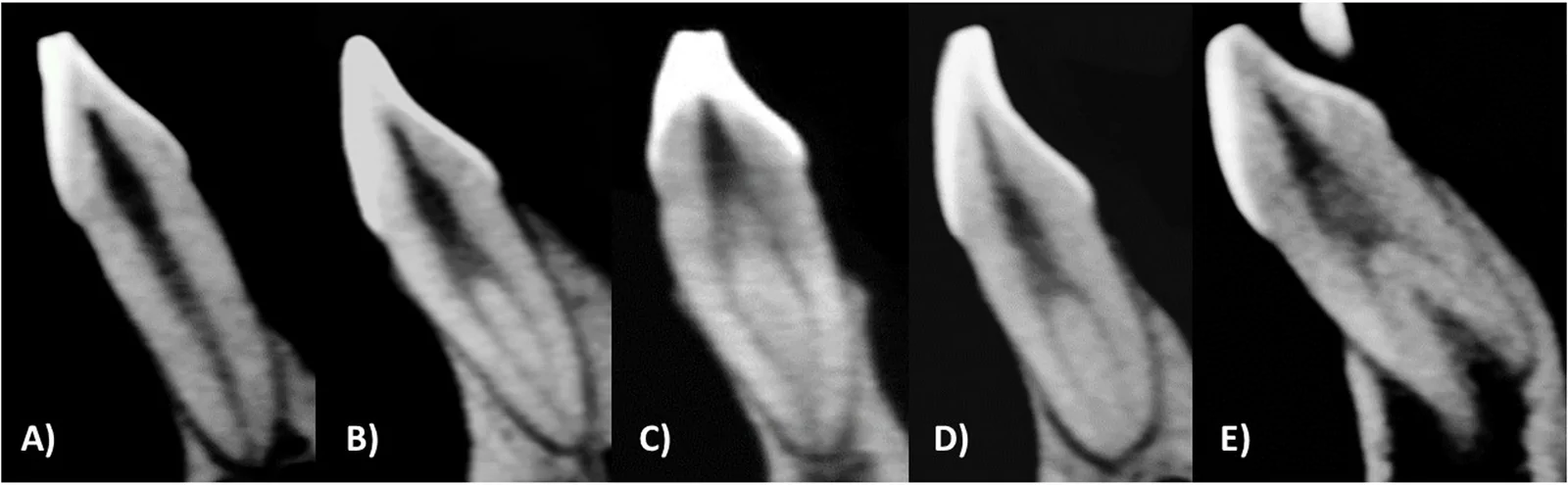
Representative sagittal CBCT images showing the root canal configurations identified in mandibular anterior teeth of the studied Chilean population according to Ahmed et al. classification. **A** 1 TN 1–1 ; (**B**) 1 TN 1-2-1; (**C**) 1 TN 2 − 1; (**D**) 1 TN 1–2; and (**E**) 2 TN B1 L1, illustrating the anatomical variability observed in the root canal system of mandibular anterior teeth

The ^1^ TN ^2−1^ configuration occurred in 11 mandibular central incisors and 11 mandibular lateral incisors (3.3% each), and in 10 mandibular canines (2.9%), while the ^1^ TN ^1–2^ configuration was recorded in 6 mandibular central incisors (1.8%), 7 mandibular lateral incisors (2.1%), and 6 mandibular canines (1.8%). Notably, mandibular canines were the only teeth to present the ^2^ TN B^1^ L^1^ configuration, with a frequency of 5 cases (1.5%) (Table 4). Fig. 3 illustrates the morphological configurations identified.

**Table 4:** Prevalence of RCS configurations according to Ahmed et al. classification

Tooth	Side	^1^ TN ^1−1^	^1^ TN ^1–2−1^	^1^ TN ^2−1^	^1^ TN ^1–2^	^2^ TN B^1^ L^1^	Total
Mandibular central incisor	Right	116 (69.0%)	45 (26.8%)	4 (2.4%)	3 (1.8%)	-	168 (100%)
	Left	117 (69.6%)	41 (24.4%)	7 (4.2%)	3 (1.8%)	-	168 (100%)
	Total	233 (69.3%)	86 (25.6%)	11 (3.3%)	6 (1.8%)	-	336 (100%)
Mandibular lateral incisor	Right	115 (68.5%)	45 (26.8%)	7 (4.2%)	1 (0.6%)	-	168 (100%)
	Left	115 (68.5%)	43 (25.6%)	4 (2.4%)	6 (3.6%)	-	168 (100%)
	Total	230 (68.4%)	88 (26.2%)	11 (3.3%)	7 (2.1)	-	336 (100%)
Mandibular canine	Right	151 (89.8%)	9 (5.4%)	3 (1.8%)	2 (1.2%)	3 (1.8%)	168 (100%)
	Left	149 (88.6%)	6 (3.6%)	7 (4.2%)	4 (2.4%)	2 (1.2%)	168 (100%)
	Total	300 (89.3%)	15 (4.5%)	10 (2.9%)	6 (1.8%)	5 (1.5%)	336 (100%)

## Discussion

Detailed knowledge of root and root canal anatomy is fundamental to the success of endodontic treatment, as morphological variations can significantly influence clinical outcomes. The present study offers population-specific data on the internal and external morphology of mandibular anterior teeth in a Chilean subpopulation, based on CBCT images analyzed using an alphanumeric coding system. By combining a three-dimensional imaging approach with a detailed classification system, this study provides a more precise representation of anatomical configurations than conventional two-dimensional methods, ensuring that subtle variations relevant to clinical practice are not overlooked.

All mandibular central and lateral incisors in this study exhibited a single root, consistent with most reports from various populations worldwide [16–19]. Although uncommon, the presence of two roots in mandibular incisors has been documented in certain populations, generally with frequencies below 1% [1, 20, 21]. Mandibular canines showed a slightly higher tendency toward this variation, with a prevalence of 1.5% in the present study—similar to that reported in a Chinese population using CBCT [19] but lower than the ~ 3% observed in Jordanian and Turkish populations [1, 20]. These rare occurrences are likely influenced by genetic, ethnic, and developmental factors, underscoring the importance of region-specific morphological data to support accurate endodontic diagnosis and treatment planning.

Regarding root length, the values obtained for mandibular central incisors, mandibular lateral incisors, and mandibular canines in this study were generally similar to those reported in other populations, such as Peruvian [22] and Brazilian [23], though slightly lower than those observed in Iranian samples [24]. Minor variations between populations may reflect ethnic, geographic, or methodological differences. Tooth length measurements also aligned closely with previous reports, including those of Bianchi et al. [25] using micro-CT, with only small discrepancies between sides and tooth types. To date, no other Chilean studies have evaluated tooth length in mandibular anterior teeth using CBCT, underscoring the relevance of the present data for clinical application in this population.

Bilateral symmetry is clinically relevant because it allows clinicians to anticipate the morphology of the contralateral tooth [26]. International studies have reported symmetry rates frequently exceeding 90% [20, 26, 27]. In the present study, symmetry frequencies were also high—above 80%—although slightly lower than some previous reports, which may be explained by differences in sample size, imaging methodology, or population-specific anatomical traits.

The most prevalent canal configuration in both mandibular central and lateral incisors in this study was ^1^ TN ^1−1^, followed by ^1^ TN ^1–2−1^, a distribution consistent with previous findings across different populations (Table 5) [1, 10, 16, 28]. Mandibular canines predominantly exhibited the ^1^ TN ^1−1^ configuration, as also reported in other international studies (Table 6) [1, 10, 29, 30]. Less common but clinically relevant configurations—including ^1^ TN ^1–2−1^, ^1^ TN ^2−1^, and ^2^ TN B^1^ L^1^—were also identified, highlighting the advantage of the alphanumeric coding system in capturing complex anatomical variations that older systems, such as Vertucci’s, cannot fully describe. Recognizing these patterns broadens the understanding of root canal anatomy in the Chilean population and reinforces the clinical value of adopting comprehensive, updated classification systems. From a clinical perspective, the identification of canal configurations such as ^1^ TN ^1–2−1^ and ^1^ TN ^2−1^ is particularly relevant, as these anatomical patterns involve canal bifurcation and/or merging, potentially increasing the complexity of canal location, negotiation, cleaning, shaping, and obturation procedures. Failure to recognize these variations may result in untreated canal spaces, which could adversely affect treatment outcomes. Similarly, although uncommon, two-rooted mandibular canines may present additional challenges during access cavity preparation and canal identification. Therefore, when clinically justified, CBCT may serve as a valuable adjunct for the preoperative detection of complex anatomical configurations, thereby reducing the likelihood of missed canals and facilitating more predictable endodontic treatment.

**Table 5:** Summary of CBCT-Based Studies Reporting Mandibular Incisor Root Canal Morphology Using Ahmed et al.'s Classification System and Included in the Discussion

Author	Population	Study design	Sample size	Ahmed et al. classification for mandibular central incisors	Ahmed et al. classification for mandibular lateral incisors
Taha et al. (2024) [1]	Jordanian	Cross-sectional cohort	Mandibular central incisor: 1114 Mandibular lateral incisor: 1114	^1^ TN ^1−1^: 787 (70.6%) ^1^ TN ^1–2−1^: 300 (26.9%) ^1^ TN ^2−1^: 8 (0.7%) ^1^ TN ^1–2^: 3 (0.3%) ^1^ TN ^2−1−2−1^: 16 (1.4%)	^1^ TN ^1−1^: 779 (69.9%) ^1^ TN ^1–2−1^: 311 (27.9%) ^1^ TN ^2−1^: 7 (0.6%) ^1^ TN ^1–2^: 3 (0.3%) ^1^ TN ^2−1−2−1^: 11 (1%) ^2^ TN B^1^ L^1^: 3 (0.3%)
Buchanan et al. (2022) [16]	South African	Cross-sectional cohort.	Mandibular central incisor: 387 Mandibular lateral incisor: 387	^1^ TN ^1−1^: 245 (63.3%) ^1^ TN ^1–2−1^: 129 (33.3%) ^1^ TN ^2−1^: 9 (2.4%) ^1^ TN ^1–2^: 2 (0.5%) ^1^ TN ^2−1−2^: 1 (0.25%) ^1^ TN ^1–2−1–2^: 1 (0.25%)	^1^ TN ^1−1^: 256 (66.1%) ^1^ TN ^1–2−1^: 115 (29.7%) ^1^ TN ^2−1^: 8 (2.1%) ^1^ TN ^1–2^: 5 (1.3%) ^1^ TN ^1–2−1–2^: 3 (0.8%)
Iqbal et al. (2022) [28]	Saudi	Cross-sectional cohort	Mandibular central incisor: 570 Mandibular lateral incisor: 570	^1^ TN ^1−1^: 489 (85.8%) ^1^ TN ^1–2−1^: 74 (13%) ^1^ TN ^1–2^: 7 (1.2%)	^1^ TN ^1−1^: 391 (68.6%) ^1^ TN ^1–2−1^: 144 (25.3%) ^1^ TN ^1–2^: 35 (6.1%)
Karobari et al. (2020) [10]	Malaysian	Cross-sectional cohort	Mandibular central incisor: 1692 Mandibular lateral incisor: 1701	^1^ TN ^1−1^: 1104 (65.2%) ^1^ TN ^1–2−1^: 521 (30.8%) ^1^ TN ^2−1^: 20 (1.2%) ^1^ TN ^1–2^: 20 (1.2%) ^1^ TN ^2^: 2 (0.1%) ^1^ TN ^2−1−2^: 2 (0.1%) ^1^ TN ^1–2−1–2^: 6 (0.4%) ^1^ TN ^2−1−2−1^: 17(1%)	^1^ TN ^1−1^: 763 (44.9%) ^1^ TN ^1–2−1^: 870 (51.1%) ^1^ TN ^2−1^: 18 (1.1%) ^1^ TN ^1–2^: 30 (1.8%) ^1^ TN ^2^: 4 (0.2%) ^1^ TN ^1–2−1–2^: 3 (0.2%) ^1^ TN ^2−1−2−1^: 13 (0.8%)

**Table 6:** Summary of CBCT-Based Studies Reporting Mandibular Canine Root Canal Morphology Using Ahmed et al.'s Classification System and Included in the Discussion

Author	Population.	Study design.	Sample size.	Ahmed et al. classification for mandibular canines.
Taha et al. (2024) [1]	Jordanian	Cross-sectional cohort	Mandibular canine: 1114	^1^ TN ^1−1^: 972 (87.3%) ^1^ TN ^1–2−1^: 77 (6.9%) ^1^ TN ^2−1^: 22 (2%) ^1^ TN ^1–2^: 4 (0.4%) ^2^ TN B¹ L¹: 35 (3.1%) ^1^ TN ^2−1−2−1^: 4 (0.4%)
Shrestha et al. (2024) [30]	Nepalese	Cross-sectional cohort	Mandibular canine: 741	^1^ TN ^1−1^: 634 (85.6%) ^1^ TN ^1–2−1^: 26 (3.5%) ^1^ TN ^2−1^: 34 (4.6%) ^1^ TN ^2−2^: 19 (2.5%) ^1^ TN ^1–2^: 28 (3.8%)
Mirza (2022) [29]	Saudi	Cross-sectional cohort	Mandibular canine 952	^1^ TN ^1−1^: 928 (97.5%) ^1^ TN ^1–2−1^: 10 (1.1%) ^1^ TN ^2−1^: 2 (02%) ^2^ TN B¹ L¹: 12 (1.2%)
Karobari et al. (2020) [10]	Malaysian	Cross-sectional cohort	Mandibular canine: 1183	^1^ TN ^1−1^: 1062 (89.8%) ^1^ TN ^1–2−1^: 104 (8.8%) ^1^ TN ^2−1^: 3 (0.2%) ^1^ TN ^1–2^: 2 (0.1%) ^1^ TN ^1–2−1–2^: 1 (0.1%) ^1^ TN ^2−1−2−1^: 6 (0.5%) ^2^ TN B¹ L¹: 5 (0.5%)

Although the classification proposed by Ahmed et al. offers a comprehensive and standardized framework for describing root and canal morphology, its practical application in both research and clinical settings may present certain challenges. In the authors’ opinion, the high level of detail provided by the system, while advantageous for describing anatomical complexity, may render its application more time-consuming when compared with simpler classification systems, particularly when reporting teeth with multiple complex configurations. Furthermore, clinicians unfamiliar with the nomenclature may find the notation less intuitive than traditional classification systems. Therefore, although the Ahmed classification provides a highly detailed anatomical description, its routine adoption in everyday clinical practice may be more limited than in research settings.

These findings represent a significant contribution to the scientific literature, marking the first investigation of mandibular anterior teeth morphology in a Chilean population using CBCT. The substantial sample size and use of advanced imaging strengthen the reliability of the results; however, certain limitations must be acknowledged. The sample originated from a specific region in southern Chile, which may not fully reflect the anatomical variability across other Chilean regions or ethnic groups. Furthermore, although CBCT is a powerful tool for in vivo assessment of root canal morphology, its spatial resolution remains lower than that of micro-CT, potentially limiting the detection of fine anatomical details such as accessory canals and isthmuses. Future multicenter studies encompassing more diverse populations, combined with complementary high-resolution imaging methods, are recommended to validate and expand upon the present findings.

## Conclusion

In this Chilean subpopulation, mandibular incisors and canines most frequently presented a single root and a single canal. A smaller proportion exhibited more complex anatomical configurations, reinforcing the need for careful assessment during endodontic diagnosis and treatment. Root and corono-radicular lengths were consistent with those reported in other populations, and the high rate of bilateral symmetry may assist clinicians in predicting contralateral morphology. CBCT proved useful for in vivo evaluation of root anatomy. 

## Data Availability

The data that support the findings of this study are available from the corresponding author upon reasonable request.

## Abbreviations

CBCT: Cone-beam computed tomography
micro-CT: Micro-computed tomography
TN: Tooth number
R: Number of roots
O: Orifice
C: Canal
F: Apical foramen
